# Mechanism of activation of SGK3 by growth factors via the Class 1 and Class 3 PI3Ks

**DOI:** 10.1042/BCJ20170650

**Published:** 2018-01-02

**Authors:** Nazma Malik, Thomas Macartney, Annika Hornberger, Karen E. Anderson, Hannah Tovell, Alan R. Prescott, Dario R. Alessi

**Affiliations:** 1 MRC Protein Phosphorylation and Ubiquitylation Unit, College of Life Sciences, University of Dundee, Dundee DD1 5EH, U.K.; 2 Signalling Programme, Babraham Institute, Babraham Research Campus, Cambridgeshire CB22 3AT, U.K.; 3 Light Microscope Facility, College of Life Sciences, University of Dundee, Dundee DD1 5EH, U.K.

**Keywords:** insulin-like growth factor, kinases, phosphoinositide 3-kinase

## Abstract

Derailment of the PI3K-AGC protein kinase signalling network contributes to many human diseases including cancer. Recent work has revealed that the poorly studied AGC kinase family member, SGK3, promotes resistance to cancer therapies that target the Class 1 PI3K pathway, by substituting for loss of Akt kinase activity. SGK3 is recruited and activated at endosomes, by virtue of its phox homology domain binding to PtdIns(3)P. Here, we demonstrate that endogenous SGK3 is rapidly activated by growth factors such as IGF1, through pathways involving both Class 1 and Class 3 PI3Ks. We provide evidence that IGF1 enhances endosomal PtdIns(3)P levels via a pathway involving the UV-RAG complex of hVPS34 Class 3 PI3K. Our data point towards IGF1-induced activation of Class 1 PI3K stimulating SGK3 through enhanced production of PtdIns(3)P resulting from the dephosphorylation of PtdIns(3,4,5)P_3_. Our findings are also consistent with activation of Class 1 PI3K promoting mTORC2 phosphorylation of SGK3 and with oncogenic Ras-activating SGK3 solely through the Class 1 PI3K pathway. Our results highlight the versatility of upstream pathways that activate SGK3 and help explain how SGK3 substitutes for Akt following inhibition of Class 1 PI3K/Akt pathways. They also illustrate robustness of SGK3 activity that can remain active and counteract physiological conditions or stresses where either Class 1 or Class 3 PI3K pathways are inhibited.

## Introduction

SGK3 (serum- and glucocorticoid-regulated kinase family member 3) belongs to the AGC family of protein kinases that orchestrate a wide range of biological responses including controlling cell growth, proliferation, metabolism and intracellular trafficking possesses [1]. Disruptions in diverse AGC kinase signalling pathways and the upstream PI3K (phosphoinositide 3-kinase) pathways that control their activity contribute to major diseases such as cancer, inflammation heart failure and diabetes [2]. Like other members of the AGC kinases including closely related Akt isoforms, SGK3 is activated following phosphorylation of its kinase domain T-loop Thr320 residue by PDK1 (3-phosphoinositide-dependent kinase-1) and phosphorylation of its C-terminal Ser422 hydrophobic motif residue by mTORC2 (mTOR complex 2) [3,4]. Like other AGC kinase members, phosphorylation of the hydrophobic residue of SGK3 by mTORC2 promotes T-loop phosphorylation and activation by PDK1 [5–7]. The two other SGK isoforms, namely SGK1 and SGK2, do not possess any phosphoinositide-binding domain and their activity is regulated by mTORC2 phosphorylating the hydrophobic motif residue, triggering activation by PDK1 [5,8,9].

SGK3 possesses an N-terminal PtdIns(3)P-binding PX (phox homology) domain that enables it to associate with endosomal membranes, where a large fraction of the cellular pool of PtdIns(3)P is located [6,10,11]. Endosomal PtdIns(3)P is generated by the Class 3 PI3K family member termed hVps34 (human vacuolar protein sorting 34) that phosphorylates PtdIns (phosphatidylinositol) to generate PtdIns(3)P [12]. PtdIns(3)P can also be generated through breakdown of PtdIns(3,4,5)P_3_ resulting from the activation of Class 1 PI3Ks [13], where PtdIns(3,4,5)P_3_ is converted into PtdIns(3)P via the sequential actions of the phosphatidylinositol 5-phosphatase (SHIP2, SH2-containing inositol 5′-phosphatase) [14] and phosphatidylinositol 4-phosphatases (INPP4A/B) [15]. PtdIns(3)P is rapidly dephosphorylated in cells to PtdIns by a family of myotubularin PtdIns 3-phosphatases [16]. Mutations that ablate PX domain PtdIns(3)P binding or treatment of cells with hVps34 inhibitors blocked SGK3 endosomal localisation and suppressing kinase activity, by lowering phosphorylation of T-loop and hydrophobic motifs [6]. Recent biochemical studies suggested that PtdIns(3)P binding to the PX domain promotes SGK3 phosphorylation of the T-loop residue by PDK1 [17].

Instead of an N-terminal PX domain, Akt kinases possess a pleckstrin homology (PH) domain that binds to the plasma membrane-localised PtdIns(3,4,5)P_3_ generated in response to agonists that trigger activation of Class 1 PI3Ks [18,19]. Akt will also bind to the immediate breakdown product of PtdIns(3,4,5)P_3_, namely PtdIns(3,4)P_2_, a reaction catalysed by SHIP2 (SH2-containing inositol 5′-phosphatase) [14,20]. Analogous to SGK3, binding of Akt to PtdIns(3,4,5)P_3_/PtdIns(3,4)P_2_ induces a conformational change that promotes phosphorylation of the Thr308 T-loop residue by PDK1 [21–23]. mTORC2-mediated phosphorylation of the Ser473 hydrophobic motif residue of Akt also promotes PDK1 phosphorylation of the T-loop residue [24]. Once activated, Akt controls critical processes such as metabolism and growth by phosphorylating dozens of key targets including FOXO (forkhead box O) transcription factors, TSC2 (tuberous sclerosis complex 2), NDRG1 (N-Myc downstream-regulated gene-1) and GSK3 (glycogen synthase kinase 3) [19].

Akt and SGK possess similar substrate specificities and can phosphorylate overlapping substrates [25–27]. A significant proportion of breast cancer cells with mutations in PTEN or Class 1 PI3K are inherently resistant to inhibitors of PI3K or Akt, as they express high levels of SGK1 that can substitute for Akt [27,28]. In human clinical trials, long-term administration of a Class 1 PI3Kα inhibitor (BYL719) resulted in metastatic tumours in some patients that became resistant to the inhibitor by up-regulating SGK1 [28]. In another study, prolonged treatment of breast cancer cell lines with Class 1 PI3K or Akt inhibitors induced resistance, by increasing the expression and activation of SGK3 [17]. The treatment of BT-474 breast cancer cell-derived tumours in a xenograft model with a combination of SGK and Akt inhibitors induced greater tumour regression than achieved through the administration of either inhibitor alone [17].

In the present study, we investigate how the activity of endogenous SGK3 is controlled. We find that SGK3 is rapidly stimulated by growth factors through a pathway that involves both Class 1 and Class 3 PI3Ks. Our data are consistent with a model, in which growth factors stimulate SGK3 activity by enhancing endosomal PtdIns(3)P production via the UV-RAG complex of hVPS34 and through a pathway involving SHIP2-controlled metabolism of PtdIns(3,4,5)P_3_ produced by Class 1 PI3K, to PtdIns(3)P. These findings highlight the adaptability of the mechanisms controlling activation of SGK3.

## Materials and methods

### Materials

[γ-^32^P]ATP was from PerkinElmer. Triton X-100, EDTA, EGTA, sodium orthovanadate, sodium glycerophosphate, sodium fluoride, sodium pyrophosphate, 2-mercaptoethanol, sucrose, benzamidine, Tween 20, Tris–HCl, sodium chloride, magnesium acetate and doxycycline were from Sigma. PMSF was from Melford. Protein G Sepharose was from GE Healthcare. Tissue culture reagents, Novex 4–12% Bis–Tris gels and NuPAGE LDS sample buffer were from Invitrogen (NP0008). Polyethylenimine was from Polysciences. Polybrene was from Sigma. Ampicillin was from Merck. P81 phosphocellulose paper was from Whatman. Methanol and chloroform were from VWR Chemicals. 1,2-dioleoyl-*sn*-glycero-3-phosphoinositol was from Avanti Polar Lipids. Inhibitors GDC-0941 (Axon Medchem) and MK-2206 (Selleck) were purchased from the indicated suppliers. VPS34-IN1 (1-[[2-[(2-chloropyridin-4yl)amino]-4′-(cyclopropylmethyl)-[4,5′-bipyrimidin]-2′-yl]amino]-2-methyl-propan-2-ol) was synthesised as described in patent WO 2012085815 A1 as described before [6]. The Sanofi compound 14 h was synthesised as described recently [29]. Plasmids used in the present study were generated by the MRC-PPU reagents and Services team (https://mrcppureagents.dundee.ac.uk/) including HA-SHIP2 (DU27327), HA-NRAS G12D (DU52590), HA-KRAS G12C (DU26569), HA-KRAS G12D (DU26536), HA-ERBB2 V842I (DU26611) and HA-EGFR L833R (DU26542). All DNA constructs were verified by DNA sequencing, performed by the MRC-PPU DNA Sequencing and Service (http://www.dnaseq.co.uk). All constructs are available to request from the MRC-PPU reagents webpage (http://mrcppureagents.dundee.ac.uk), and the unique identifier (DU) numbers indicated above provide direct links to the cloning and sequence details. The retroviral expression system vectors pCMV-Gag-Pol and pCMV-VSVG constructs were from Clontech.

### Cell culture, transfection and cell lysis

HEK293 cells were purchased from the American Tissue Culture Collection and cultured in DMEM supplemented with 10% (v/v) foetal bovine serum, 2 mM l-glutamine, 100 U/ml penicillin and 0.1 mg/ml streptomycin. Inhibitor treatments were carried out as described in figure legends. The cells were lysed in buffer containing 50 mM Tris–HCl (pH 7.5), 1 mM EGTA, 1 mM EDTA, 1% (v/v) Triton X-100, 1 mM sodium orthovanadate, 50 mM NaF, 5 mM sodium pyrophosphate, 0.27 M sucrose, 10 mM sodium 2-glycerophosphate, 0.2 mM phenylmethylsulfonyl fluoride and 1 mM benzamidine. Lysates were clarified by centrifugation at 16 000 ***g*** for 10 min at 4°C. Protein concentration was calculated using the Bradford assay (Thermo Scientific). Immunoblotting and immunoprecipitation were performed using standard procedures. The signal was developed using the ECL Western Blotting Detection Kit (Amersham) on Amersham Hyperfilm ECL film (Amersham).

### Antibodies

The following antibodies were raised in sheep, by the MRC-PPU reagents and Services team (https://mrcppureagents.dundee.ac.uk/) and affinity-purified against the indicated antigens: anti-Vps34 (S672B; third bleed; raised against full-length human Vps34) (DU3303), anti-Beclin1 (S900B; first bleed; raised against full-length human Beclin1) (DU7159), anti-UV-RAG (S323D; third bleed; raised against full-length human UV-RAG) (DU 36785), anti-Akt1 (S695B, third bleed; raised against residues 466–480 of human Akt1: RPHFPQFSYSASGTA), anti-NDRG1 (S276B, third bleed; raised against full-length human NDRG1) (DU1557), anti-SGK3 (S037D, third bleed; raised against human SGK3 PX domain comprising residues 1–130 of SGK3), anti-PRAS40 (proline-rich Akt substrate 40 kDa) (S115B, first bleed; raised against residues 238–256 of human PRAS40: DLPRPRLNTSDFQKLKRKY) and anti-(phospho-PRAS40 Thr^246^) (S114B, second bleed; raised against residues 240–251 of human PRAS40: CRPRLNTpSDFQK). Anti-Vps15 was raised in rabbit (R1737; third bleed; raised against residues 433–667) (DU 39129). Anti-phospho-Akt Thr308 (#4056), anti-phospho-NDRG1 Thr346 (#5482), anti-EEA1 (early endosome autoantigen 1; #2411), anti-SIN1 (#12860), anti-GAPDH (#2118) and anti-phospho-SGK3 Thr320 (#5642) antibodies were purchased from Cell Signaling Technology. GFP-Trap® beads (gta-10) and rat anti-GFP (green fluorescent protein) antibody (3H9) were purchased from Chromotek. Secondary antibodies coupled to HRP (horseradish peroxidase) were obtained from Thermo Scientific.

### Immunoprecipitation and assay of SGK3 and Akt

*In vitro* kinase activity of SGK3 and Akt was assayed by measuring [γ-^32^P]ATP incorporation into Crosstide substrate peptide [GRPRTSSFAEGKK] [6,30]. Endogenous SGK3 or Akt was immunoprecipitated from 2 mg HEK293 cell lines using an anti-SGK3 antibody (S037D, third bleed) or an anti-Akt antibody (S695B, third bleed). Immunoprecipitates were washed in sequence with lysis buffer containing high salt concentration (500 mM NaCl), low salt concentration (150 mM) and buffer A (50 mM Tris–HCl, pH 7.5 and 0.1 mM EGTA). Reactions were carried in 40 µl of total volume containing 0.1 mM [γ-^32^P]ATP (400–1000 c.p.m./pmol), 10 mM magnesium acetate and 30 µM Crosstide peptide. Reactions were terminated by adding 10 µl of 0.1 mM EDTA and spotting 40 µl of the reaction mixture on P81 paper, which were immediately immersed into 50 mM orthophosphoric acid. Papers were washed several times in 50 mM orthophosphoric acid, rinsed in acetone and air-dried. Radioactivity was quantified by Cerenkov counting. One unit of enzyme activity was defined as the amount of enzyme that catalyses incorporation of 1 nmol of [γ-^32^P]ATP into the substrate over 1 min.

### *In vitro* kinase assays of hVPS34 and UV-RAG

Cells were treated as described in figure legends prior to lysis in NP-40 lysis buffer [50 mM HEPES (pH 7.4), 150 mM NaCl, 1 mM EDTA, 10% (v/v) glycerol and 0.5% NP-40]. Endogenous GFP-UV-RAG or GFP-Vps34 was immunoprecipitated using 10 µl GFP-Trap® beads (Chromotek) and 2 mg of clarified cell extract. The immunoprecipitates were subjected to PI3K assays essentially as described previously [31,32]. Beads were washed twice in NP-40 lysis buffer containing high salt concentration (500 mM NaCl), twice with NP-40 lysis buffer and finally twice with lipid kinase assay (LKA) buffer [10 mM MnCl_2_, 20 mM Tris (pH 7.5), 67 mM NaCl and 0.02% (w/v) CHAPS]. hVPS34 PI 3-kinase activity was assayed in a final volume of 40 µl containing 10 µg of phosphatidylinositol liposomes (bovine liver Phosphoinositide, extruded through a 100-nm filter—Avanti Mini-Extruder), 5 µM ATP and 7.5 µCi ^32^P γ-ATP in LKA buffer. Reactions were agitated for 30 min at 30°C before centrifugation through a Spin-X column to separate the ^32^P-labelled phosphatidylinositol in the flow-through and the immunoprecipitated hVPS34 complex bound to the beads. A mixture of 500 µl of methanol : chloroform : hydrocholoric acid (200 : 100 : 3.5) was added to the flow-through and 90 µl of 2× LDS sample buffer added to the beads and the resulting suspension heated at 70°C for 10 min to elute protein. To extract the ^32^P-labelled phosphatidylinositol 3-phosphate from the flow-through, 180 µl of chloroform and 300 µl of 0.1 M hydrochloric acid were added, and the samples were vortexed gently and then centrifuged at 1000 ***g*** for 1 min at room temperature. The lower lipid-containing chloroform layer was retained and dried by centrifugal evaporation. Lipids were re-dissolved in 60 µl of chloroform and spotted onto thin layer chromatography plates (Silica 60, Merck-Millipore) activated in potassium oxalate [1% (w/v) potassium oxalate, 5 mM EDTA and 50% (v/v) methanol]. Chromatography was undertaken in a solvent system comprising methanol : chloroform : water : ammonium hydroxide (47 : 60 : 11.2 : 2) to separate phosphatidylinositol substrate and ^32^P-labelled phosphatidylinositol 3-phosphate product. Once dried, the plate was subjected to autoradiography in order to visualise ^32^P-labelled phosphatidylinositol 3-phosphate.

### Ptdins(3)P PX domain staining and immunofluorescence

For selective PtdIns(3)P staining, the GST-tagged HRS 2× FYVE domain (residues 147–223) and the mutant GST-tagged 2× FYVE (residues 147–223) H176A H177A (DU47684) were expressed in *Escherichia coli* (BL21) and purified over a glutathione column using standard procedures. The recombinant wild-type protein was chemically conjugated to Alexa Fluor 488 and the mutant conjugated to Alexa Fluor 594 using the Alexa Fluor Microscale Protein Labelling Kit (# A30008) as per the manufacturer's protocol. For staining, a similar protocol that described before was followed [32]. Following treatment described in the figure legends, cells were washed once on ice with phosphate-buffered saline and glutamate buffer [25 mM HEPES (pH 7.4), 25 mM KCl, 2.5 mM Mg acetate, 5 mM EGTA, 150 mM potassium glutamate]. Coverslips were then immediately snap frozen in liquid nitrogen and thawed at room temperature for 0.5 min prior to two further washes with ice cold glutamate buffer prior to fixing by incubating cells in 3.7% (w/v) formaldehyde, 200 mM HEPES (pH 7.4). After 30 min at room temperature, fixed cells were quenched by incubating twice for 10 min in 10 mM HEPES, pH 7.4, and DMEM at room temperature. Samples were blocked by washing twice with blocking buffer [phosphate-buffered saline containing 1% (w/v) bovine serum albumin] and then incubated in this buffer for a further 30 min. Coverslips were next incubated for 1 h at room temperature with 5 µg/ml wild-type and mutant dye-labelled FYVE domain mixed together. Cells were then washed three times in blocking buffer. Coverslips were washed once more in water and mounted using ProLong Gold Antifade (ThermoFisher #P36931). For visualisation of SGK3-GFP, GFP-Vps34 and UV-RAG-GFP, the GFP signal was enhanced using a chicken anti-GFP antibody (Abcam) followed by anti-chicken secondary antibody conjugated to Alexa Fluor 488. EEA1 was co-stained with anti-EEA1 antibody and secondary anti-rabbit antibody conjugated to Alexa Fluor 594. Prior to staining, cells were fixed with 4% (v/v) paraformaldehyde and permeabilised with 1% (v/v) NP-40. The images were collected on an LSM710 laser scanning confocal microscope (Carl Zeiss) using the ×63 Plan-Apochromat objective (NA 1.4), using a pinhole chosen to provide a uniform 0.8 um optical section thickness in all the fluorescence channels. Images from the microscope were imported into Volocity image processing software (PerkinElmer) and batch-processed using the same custom written programmes for all the images in an experimental group. For example, in each image, endosomes were identified from the EEA1 antibody staining, and the intensity of the GFP-tagged protein in these objects was collected as the sum of the pixel intensities, normalised for the number of cells in each image (counted from the number of DAPI nuclei). The graphs show the sum of the GFP intensity per cell in arbitrary units with variation presented as SEM for the 15 images collected from each slide. Each treatment was repeated three times and graphs shown are from representative experiments.

### Generation of SGK3, SHIP2 and Sin1 knock-out cell lines and GFP-Vps34, UV-RAG-GFP and SGK3-GFP knock-in cell lines using Crispr/Cas9 genome editing

A modified Cas9 nickase system [33] was used for the generation of N-terminal GFP-VPS34, C-terminal GFP-UV-RAG and C-terminal SGK3 knock-in mutation. Optimal sgRNA pairs were identified [as close as possible to point of GFP insertion, with a low combined off-targeting score; (VPS34-sgRNA1: GCTACATCTATAGTTGTGACC (DU52071); sgRNA2: GCCCCATCGCACCGTCTGCAA (DU52082); UV-RAG-sgRNA1: GCACTTATCGGAACTCCTGCG (DU54207); sgRNA2: GCAGGTCAACAGTAGGACTG (DU54212); SGK3-sgRNA1: GCATCATCTGCCTCCAATACAC (DU48560)); sgRNA2: GAGGCAGATGATGCATTCGT (DU48555)]. Complementary oligos with *Bbs*I compatible overhangs were designed for each, annealed and the dsDNA guide inserts ligated into *Bbs*I-digested target vectors; the antisense guides (sgRNA2) were cloned onto the spCas9 D10A-expressing pX335 vector (Addgene plasmid no. 42335) and the sense guides (sgRNA1) into the puromycin-selectable pBABED P U6 plasmid (Dundee-modified version of the original Cell Biolabs pBABE plasmid). Donor constructs (VPS34-DU52175, UV-RAG-DU54245 and SGK3-DU48103) consisting of GFP flanked by ∼500 bp homology arms were synthesised by GeneArt (Life Technologies); each donor was engineered to contain sufficient silent mutations to prevent recognition and cleavage by Cas9 nuclease. sgRNA pairs for the SGK3 knock-out were as follows: SGK3-sgRNA1: GTTTTGGACTGTCCATTTGA (DU48668); sgRNA2: GTCAGATCCATCTGAAGATG (DU48667) and for SHIP2 KO as follows: sgRNA1: GCGTGTGGATGGCTGCGGAGC (DU52294); sgRNA2: GGCCAAGACCATCCCCGTGC (DU52275). sgRNA pairs for the SIN1 knock-out were as follows: sgRNA1: GTGCTGGCAGTATACAAGCGA (DU52278); sgRNA2: GCCCGATCAGGTCCTGCACCC (DU52297). HEK293 knock-out cell lines were obtained via transfection using 1 µg each of appropriate guide pairs (pBABED-Puro-sgRNA1 and pX335-CAS9-D10A-sgRNA2), knock-in cell lines were generated using guide plasmids and an additional 3 µg of donor plasmid. Sixteen hours post-transfection, cell selection was carried out using 2 µg/ml puromycin for 2 days. Transfection and selection were repeated prior to single-cell sorting by FACS. Single cells were plated in individual wells of 96-well plates and viable clones were expanded. Loss of protein in knock-out clones or integration of GFP at the target locus for knock-in clones was verified by western blotting and genomic DNA sequencing of the targeted locus.

### Affinity-directed protein missile-mediated knock-down of hVPS34 and UV-RAG

The cDNAs encoding the nanobodies aGFP16-DU54238 and VHL-aGFP16 (DU54294) were cloned into pBABED-Puro vectors (Cell Biolabs, modified) for constitutive expression as described recently [34]. Target cells were retrovirally transduced. Briefly, for retrovirus production, pBABED retroviral plasmids (6 µg), encoding appropriate proteins, were co-transfected with pCMV-gag-pol (4 µg) and pCMV-VSV-G (2 µg) in a 10-cm diameter dish of 70% confluent 293-FT cells. Plasmids were mixed in 0.6 ml Opti-MeM (Life Technologies), to which 24 µl of 1 mg/ml polyethylenimine was added. Following a 15 min incubation at room temperature, the mixture was applied dropwise to 293-FT cells. The medium was replaced 16 h post-transfection with fresh medium, and retrovirus was collected in the growth medium 24 h later following filtration through 0.45 µm filters. Target cells (∼60% confluent) were infected with the optimised titre of retrovirus medium supplemented with 10 µg/ml polybrene for 24 h prior to selection with 2 µg/ml puromycin.

### Mass spectrometry measurements of inositol lipids

Mass spectrometry was used to measure inositol lipid levels essentially as previously described [35,36], using a QTRAP 4000 (AB Sciex) mass spectrometer and employing the lipid extraction and derivatisation method described for cultured cells, with the modification that 10 ng of C17:0/C16:0 PtdIns(3,4,5)P_3_ internal standard (ISD) and 10 ng of C17:0/C16:0 PtdIns ISD were added to primary extracts, and that final samples were dried in a speed vac concentrator rather than under N2. Measurements were conducted on triplicate samples per condition. A PtdIns(3,4,5)P_3_ response ratio was calculated by dividing the PtdIns(3,4,5)P_3_ response area by the response area of PtdIns(3,4,5)P_3_ ISD in each sample. PtdIns(3,4,5)P_3_ responses were normalised to PtdIns (PI) response ratio to account for any cell input variability.

### Statistical analysis

All experiments in the present study were performed at least twice and similar results were obtained. Data were analysed using one-way ANOVA followed by Bonferroni's *post hoc* test (*P *< 0.05) using Prism software. The error bars indicate SEM.

## Results

### Activation of SGK3 by insulin-like growth factor 1

**Figure 1.**
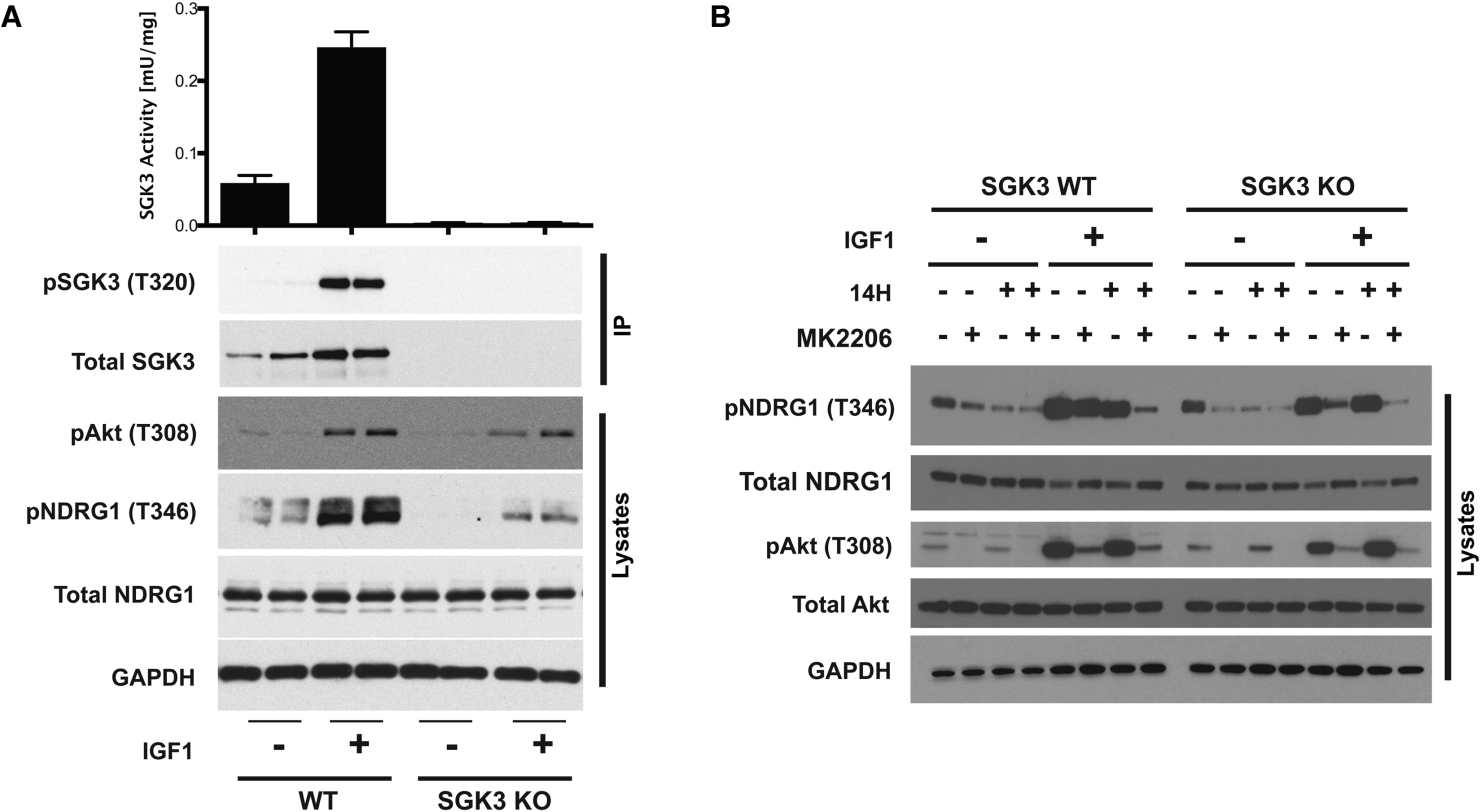
SGK3 is activated by IGF1. (**A**) Wild-type (WT) and SGK3 knock-out (KO) HEK293 cells were serum-starved for 16 h prior to stimulation with or without 50 ng/ml IGF1 for 10 min. Subsequently, cells were lysed and endogenous SGK3 was immunoprecipitated with anti-SGK3 antibody. Immunoprecipitates were assayed for SGK3 kinase activity by measuring phosphorylation of the Crosstide substrate peptide in the presence of 0.1 mM ^32^PγATP in a 30 min reaction. The lysates and immunoprecipitates were subjected to immunoblot analysis with the indicated antibodies following the kinase assay. Kinase reactions are presented as means ± SEM for triplicate reactions. Similar results were obtained in at least two independent experiments. (**B**) As in (**A**), except cells were treated with or without 3 µM 14H and/or 1 µM MK2206 for 1 h before stimulation with or without 50 ng/ml IGF1 for 10 min. Cell lysates were subjected to immunoblot analysis with the indicated antibodies.

Stimulating serum-starved HEK293 cells with insulin-like growth factor (IGF1, 50 ng/ml) for 10 min markedly enhanced endogenous SGK3 kinase activity and its T-loop phosphorylation (Figure 1A). No SGK3 activity or T-loop phosphorylation was observed in SGK3 knock-out HEK293 cells analysed in parallel, validating the assay approach (Figure 1A). Consistent with previous work showing that both Akt and SGK phosphorylate NDRG1 at Thr346 [27], a combined treatment with MK-2206 Akt inhibitor [37] and 14 h SGK inhibitor [17,29] was required to fully suppress NDRG1 phosphorylation in wild-type HEK293 cells (Figure 1B). In contrast, in SGK3 knock-out cells, a treatment with MK-2206 inhibitor alone suppressed most of the NDRG1 phosphorylation (Figure 1B). Interestingly, we note that addition of 14 h and MK2206 to SGK3 knock-out cells reduces the low levels of NDRG1 phosphorylation further compared with MK2206 alone. We speculate that this may be due to low levels of SGK1 activity that is still present in these cells [9] .

### Class 1 and Class 3 PI3Ks mediate activation of SGK3 by IGF1

**Figure 2.**
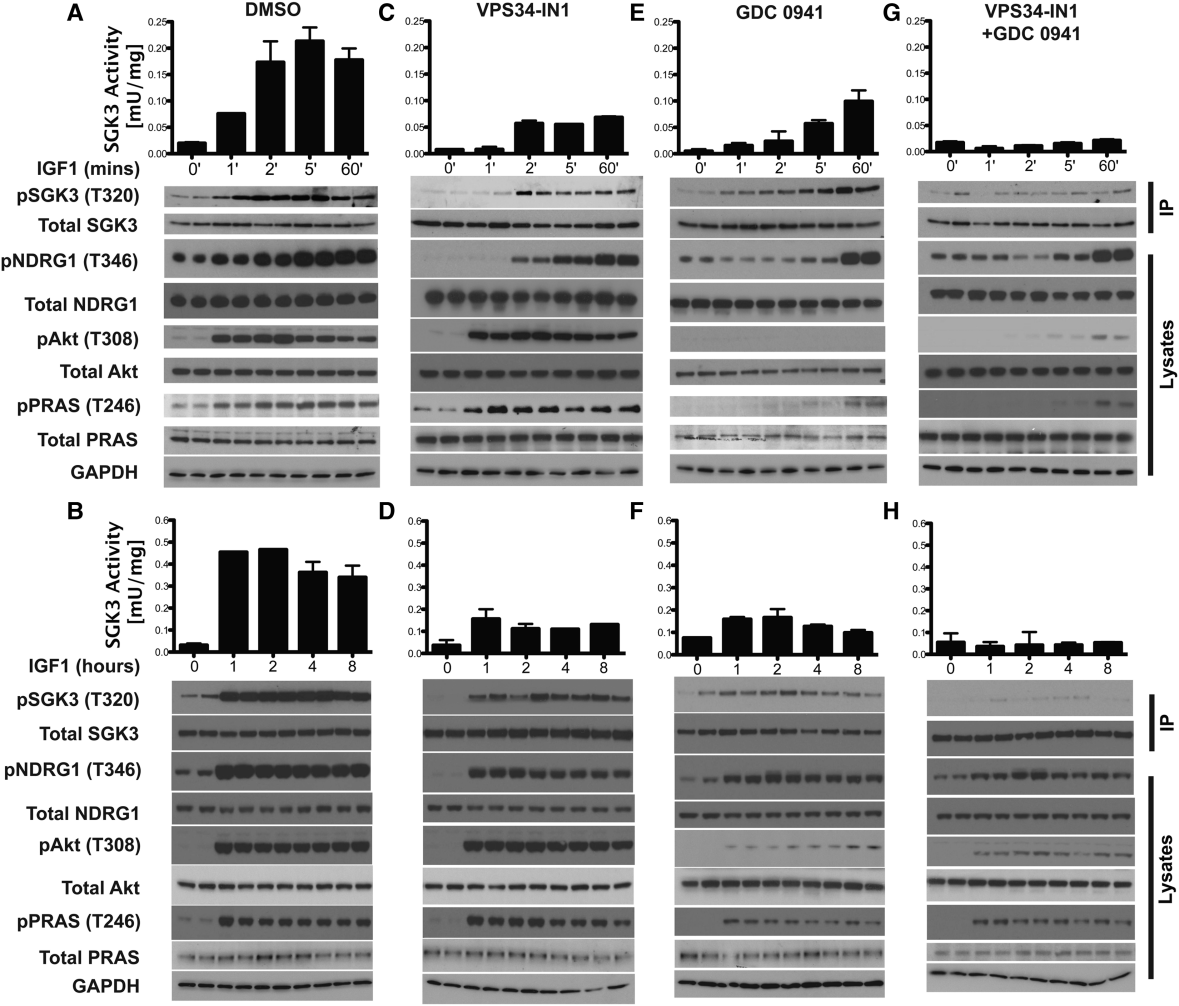
Class 1 and Class 3 PI3Ks mediate IGF1-induced SGK3 activity. HEK293 cells were serum-starved for 16 h. One  hour prior to stimulation, cells were left untreated (**A** and **B**) or treated with 1 µM VPS34-IN1 (**C** and **D**) or 0.5 µM GDC-0941 (**E** and **F**) or 1 µM VPS34-IN1 and 0.5 µM GDC-0941 (**G** and **H**). Cells were then stimulated with 50 ng/ml IGF1 for the indicated times in the presence of inhibitors. Cells were lysed and endogenous SGK3 was immunoprecipitated with anti-SGK3 antibody. The lysates and immunoprecipitates were subjected to immunoblot analysis with the indicated antibodies, after being assayed for SGK3 kinase activity by measuring phosphorylation of the Crosstide substrate peptide in the presence of 0.1 mM ^32^PγATP in a 30 min reaction. Kinase reactions are presented as means ± SEM for triplicate reactions. Similar results were obtained in at least two independent experiments for all data shown.

Time course analysis revealed that IGF1 increased SGK3 activity from a low basal level ∼3- to 4-fold within 1 min and 10-fold within 2–5 min. SGK3 remained at a high level for up to 8 h (Figure 2A,B). Treatment with the Class 3 PI3K inhibitor VPS34-IN1, which does not inhibit Class 1 PI3K [6], blocked the stimulation of SGK3 activity and NDRG1 phosphorylation observed after 1 min IGF1. Between the 2 min to 8 h IGF1 stimulation, VPS34-IN-1 treatment reduced SGK3 activation ∼4-fold (Figure 2C,D). Consistent with VPS34-IN1 not inhibiting Class 1 PI3K, Akt and its selective PRAS40 substrate were normally phosphorylated in IGF1-treated cells in the presence of VPS34-IN1 (Figure 2C,D).

In contrast, the Class 1 PI3K inhibitor GDC-0941, which does not inhibit hVPS34 [38], had no effect on basal SGK3 activity or NDRG1 phosphorylation (Figure 2E,F). After IGF1 stimulation, GDC-0941 appears to slow the rate and extent of activation of SGK3 and NDRG1 phosphorylation. At the 1–8 h time points, SGK3 activation is ∼2-fold lower (Figure 2E,F). A marked increase in NDRG1 phosphorylation in the presence of GDC-0941 was only noted at and after the 60 min time point. Treatment with VPS34-IN1 and GDC-0941 together blocked IGF1 stimulation of SGK3 activity at all time points (Figure 2G,H). We noted a moderate stimulation of NDRG1 phosphorylation at 60 min time point in the presence of both VPS34-IN1 and GDC-0941, indicating that another NDRG1 kinase may become slowly activated under these conditions (Figure 2G,H).

### SGK3 is activated by insulin and EGF

**Figure 3.**
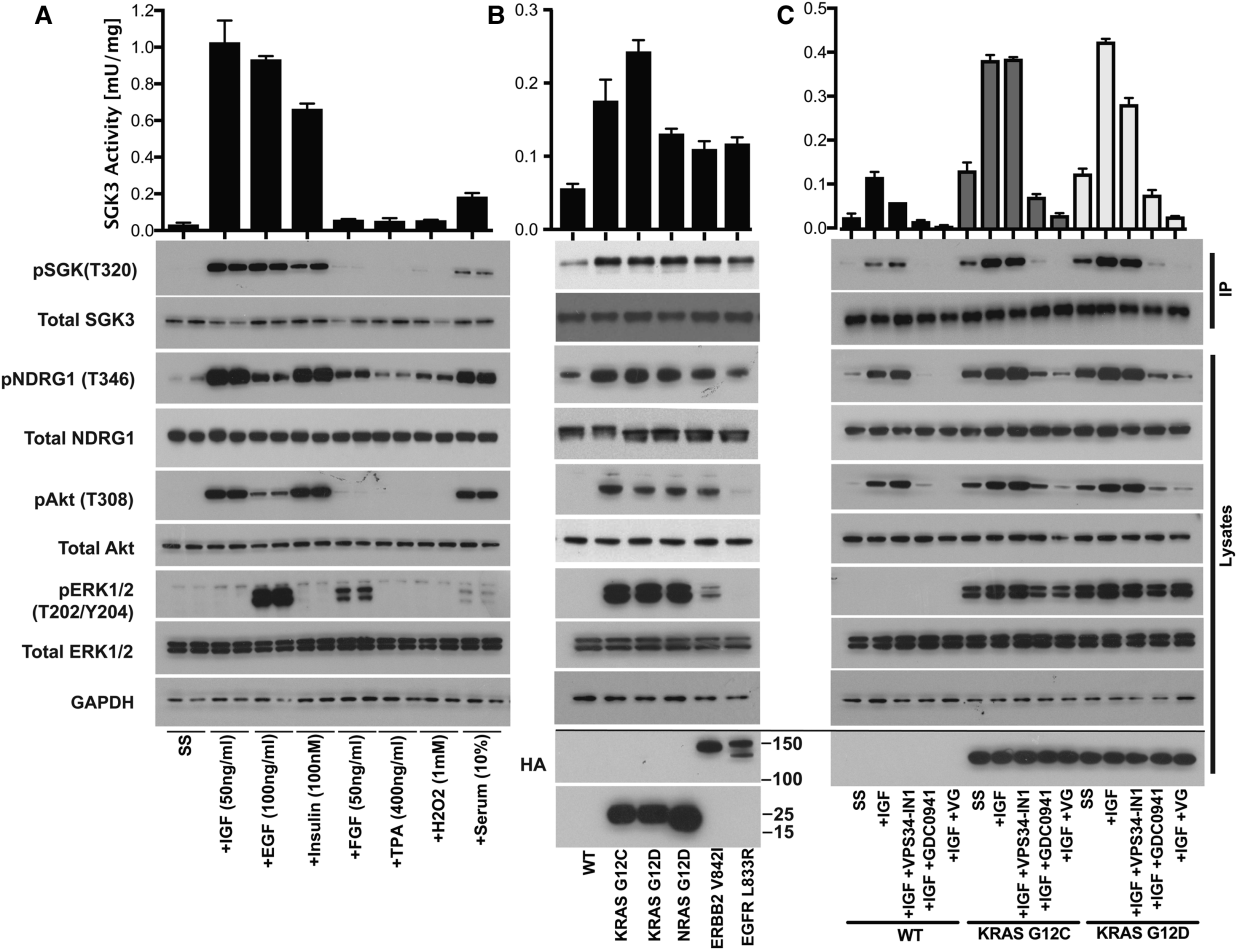
SGK3 activity is induced by growth factors, insulin and oncogenic Ras. (**A**) HEK293 cells were serum-starved for 16 h prior to stimulation with or without the indicated concentrations of IGF1, EGF, insulin, FGF, TPA, H_2_O_2_ and serum for 15 min. Endogenous SGK3 was immunoprecipitated from the lysates and SGK3 kinase activity was assessed by measuring phosphorylation of the Crosstide substrate peptide in the presence of 0.1 mM ^32^PγATP in a 30 min reaction. Both immunoprecipitates and lysates were subjected to western blotting with the indicated antibodies. (**B**) As in (**A**), except that HEK293 cells were transiently transfected when 60% confluent, with KRAS[G12C], KRAS[G12D], NRAS[G12D], ERBB2[V842I] and EGFR[L833R] (all HA epitope tagged). Forty eight hours later, WT and transfected cells were serum-starved for 16 h before cells lysed without further stimulation. (**C**) As in (**B**), except that HEK293 cells were transiently transfected with KRAS[G12C] and KRAS[G12D] and treated as indicated with VPS34-IN1 (1 µM) and/or GDC-0941 (0.5 µM) prior to stimulation with 50 ng/ml IGF1 for 10 min. Kinase reactions are presented as means ± SEM for triplicate reactions. Similar results were obtained in at least two independent experiments for all data shown.

Stimulation of HEK293 cells with insulin (100 nM), epidermal growth factor (EGF, 100 ng/ml) and serum (10% [v/v]) induced a 3–20-fold stimulation of SGK3 (Figure 3A). In contrast, FGF_basic_ (50 ng/ml), tissue plasminogen activator (400 ng/ml) or H_2_O_2_ (1 mM) failed to significantly activate SGK3 at a 15 min time point despite inducing a low level of phosphorylation of NDRG1. FGF_basic_ also induced a marked activation of ERK1/ERK2 (Figure 3A).

### Oncogenic Ras, ERBB2 and EGFR activate SGK3

We transiently transfected HEK293 cells with KRAS[G12C], KRAS[G12D], NRAS[G12D], ERB2[V842I] and EGFR[L833R] oncogenes under conditions where 80% of cells were transfected and measured the effect that this had on endogenously expressed SGK3. This revealed that all oncogenes tested, stimulated SGK3 activity 2–5-fold and enhanced NDRG1, Akt and ERK phosphorylation (Figure 3B). Strikingly, VPS34-IN1 had no significant effect on SGK3 activation or NDRG1 phosphorylation induced by overexpression of oncogenic KRAS, which was instead blocked by GDC-0941 (Figure 3C). This suggests that oncogenic KRAS is activating SGK3 through the PI3K pathway rather than hVPS34.

### IGF1 enhances endosomal PtdIns(3)P

**Figure 4.**
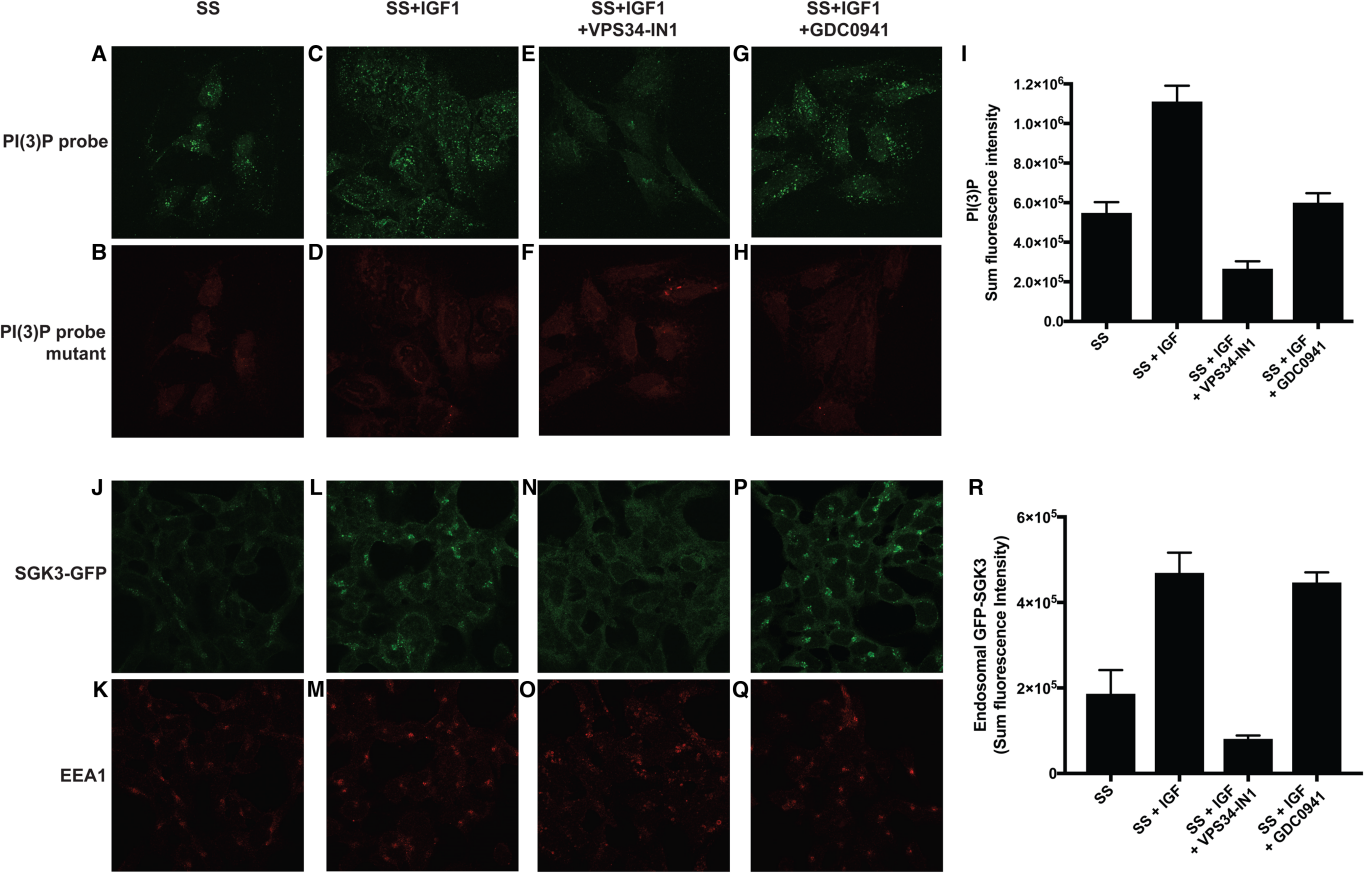
IGF1 stimulation enhances endosomal PtdIns(3)P and recruitment of GFP-SGK3 to the endosomes. HEK293 cells were serum-starved overnight then treated as indicated with or without VPS34-IN1 (1 µM) and/or GDC-0941 (0.5 µM) for 60 min prior to stimulation with 50 ng/ml IGF1 for 15 min. Cells were subsequently fixed with 4% (v/v) paraformaldehyde and stained with recombinant wild-type (WT) PtdIns(3)P-binding probe FYVE domain of HRS[147–223] conjugated to Alexa Fluor 488 conjugate (green channel) (**A**, **C**, **E** and **G**) or with the mutant non-PtdIns(3)P-binding FYVE domain of HRS[147–223, H176A/H177A] (red channel, Alexa 594). (**B**, **D**, **F** and **H**). (**I**) The histogram displays a representative quantitation of the sum fluorescence intensity of PX domain staining on endosomal structures ± SEM (increase in PtdIns(3)P levels upon IGF1 stimulation ranged from 30 to 50% compared with serum-starved cells). (**J–Q**) GFP-SGK3 knock-in HEK293 cells were serum-starved overnight and treated as above prior to IGF1 stimulation. Cells were fixed with 4% (v/v) paraformaldehyde and GFP distribution was visualised using chicken anti-GFP primary and anti-chicken Alexa Fluor 488 to enhance the GFP signal (upper panel). SGK3 co-localisation with the early endosomal marker EEA1 was visualised using rabbit anti-EEA1 primary and anti-rabbit Alexa Fluor 594 secondary antibody (lower panel). (**R**) The histogram displays a representative quantitation of the sum of intensity of fluorescent signal from GFP-SGK3, co-localising with the EEA1 marker at the endosomes ± SEM following the various treatments (increase in GFP-SGK3 levels at the endosomes ranged from 30 to 60% upon the addition of IGF1 compared with serum-starved conditions).

To assess intracellular levels of PtdIns(3)P, we used a previously described method [32], in which cells were fixed with formaldehyde and then exposed to a combination of recombinant wild-type PtdIns(3)P-binding region of the HRS2xFYVE conjugated to Alexa Fluor 488 (green dye labelled) and the non-PtdIns(3)P-binding point mutant of this FYVE domain conjugated to Alexa Fluor 594 (red dye labelled). The levels of green and red probe remaining associated with cell structures resembling endosomes after washing were used to assess endosomal levels of PtdIns(3)P (Figure 4). These experiments revealed that in wild-type serum-starved cells consistent with recent work [32], the wild-type PtdIns(3)P-binding HRS2xFYVE probe was associated with punctate structures resembling endosomes, while localisation of the non-PtdIns(3)P-binding probe was more diffuse and consistent with background binding (Figure 4, compare upper and lower panels). We observed 10 min IGF1 stimulation increased ∼2-fold the number of punctate structures in three separate experiments (compare Figure 4A,C,I). Treatment with VPS34-IN1 had a marked effect and reduced the number of punctate structures and the amount of staining associated with each cell ∼3-fold (Figure 4E,I), only GDC-0941 had a moderate effect on reducing binding of the PtdIns 3P-probe to endosome structures (Figure 4G,I). IGF1 stimulation had no effect on binding of the mutant probe in the red channel (Figure 4B,D,F,H).

### IGF1 enhances endosomal localisation of SGK3 via hVPS34

To investigate localisation of endogenous SGK3 in HEK293 cells, we deployed a CRISPR/CAS9 approach to homozygously knock-in a GFP tag onto the C-terminus of SGK3. The resultant fusion protein is expressed at a similar level to non-tagged SGK3 in wild-type cells (Supplementary Figure S1A). GFP immunofluorescence analysis of serum-starved cells revealed that a significant proportion of SGK3-GFP was co-localised with the EEA1 endosomal marker and located in punctate structures (Figure 4J,K). Stimulation with IGF1 for 10 min induced an ∼2-fold increase in endosomal localisation of SGK3-GFP with EEA1 in three independent experiments (Figure 4L,M,R). Treatment with VPS34-IN1 significantly reduced endosomal localisation of SGK3-GFP (Figure 4N,O,R). The structurally unrelated hVPS34 inhibitor, termed SAR405 [39], also blocked endosomal localisation of SGK3-GFP (Supplementary Figure S1B). GDC-0941 had no significant effect on SGK3-GFP endosomal localisation (Figure 4P,Q,R).

### IGF1 does not affect endosomal localisation or activity of hVPS34

**Figure 5.**
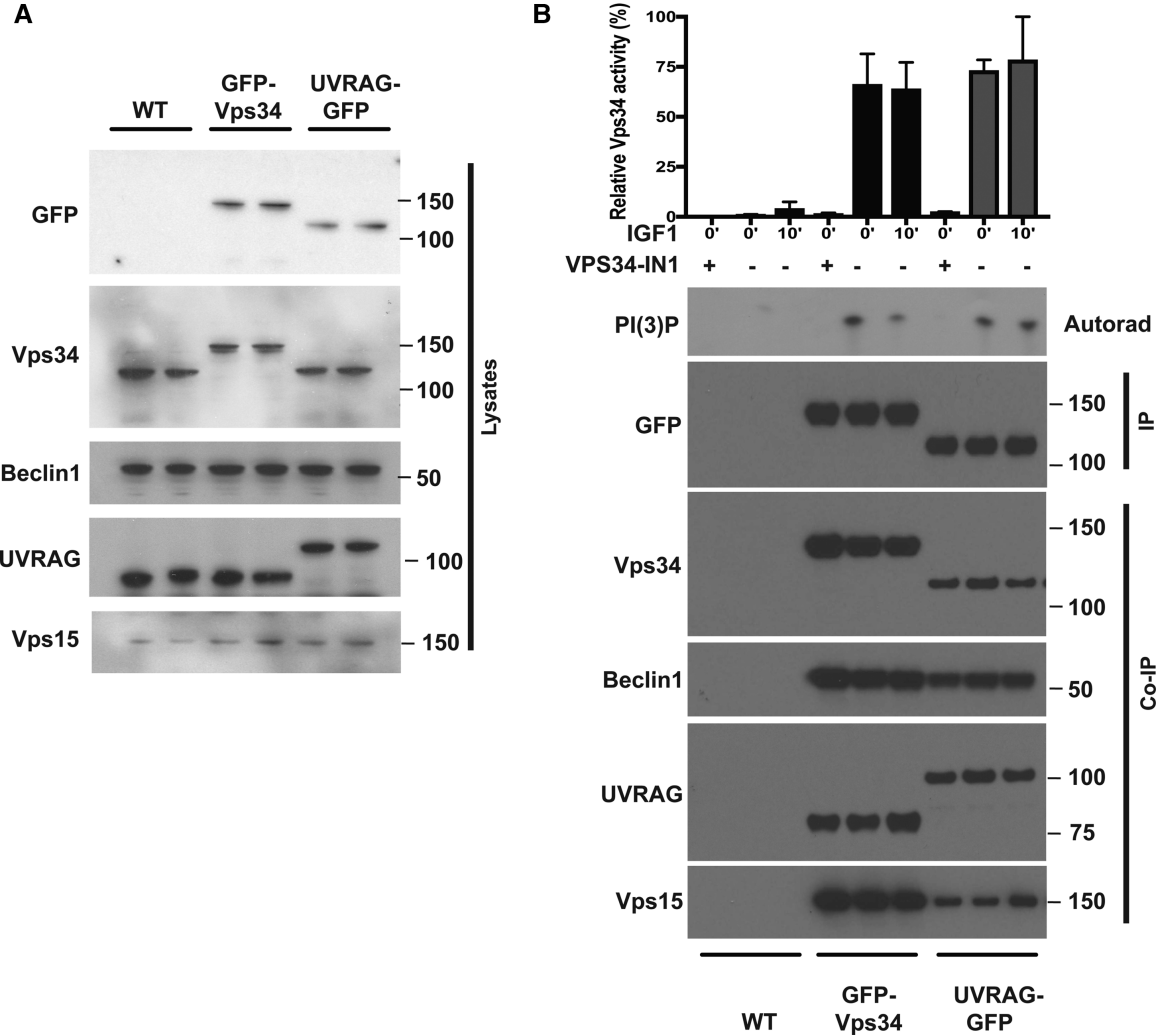
IGF1 does not stimulate hVPS34 activity. Wild-type (WT), GFP-Vps34 or GFP-UV-RAG knock-in HEK293 cells were cultured in serum-lysed and subjected to immunoblot analysis with the indicated antibodies. (**B**) WT, GFP-Vps34 or GFP-UV-RAG knock-in HEK293 cells were serum-starved overnight prior to treatment with or without 50 ng/ml IGF1 for 15 min. Cells were subsequently lysed using a 1% (v/v) NP-40 lysis buffer and immunoprecipitations undertaken using GFP-TRAP antibodies. The immunoprecipitates were subjected to a kinase assay in the presence of PtdIns (5 µg) and 0.1 mM ^32^PγATP for a 30 min reaction measure in the absence or presence of VPS43-IN1 (1 µM) as indicated. Reactions were chromatographed on a Silica 60 TLC plate and ^32^P-radioactivity associated with the spot, corresponding to PtdIns(3)P, was visualised by autoradiography and quantified by Cerenkov scintillation. Immunoprecipitates were also subjected to immunoblot analysis with the indicated antibodies. In the top panel, we show the quantitation of the VPS34 PI 3-kinase activity. The data are presented as means ± SEM for triplicate reactions. Similar results were obtained in at least two independent experiments for all data shown.

**Figure 6.**
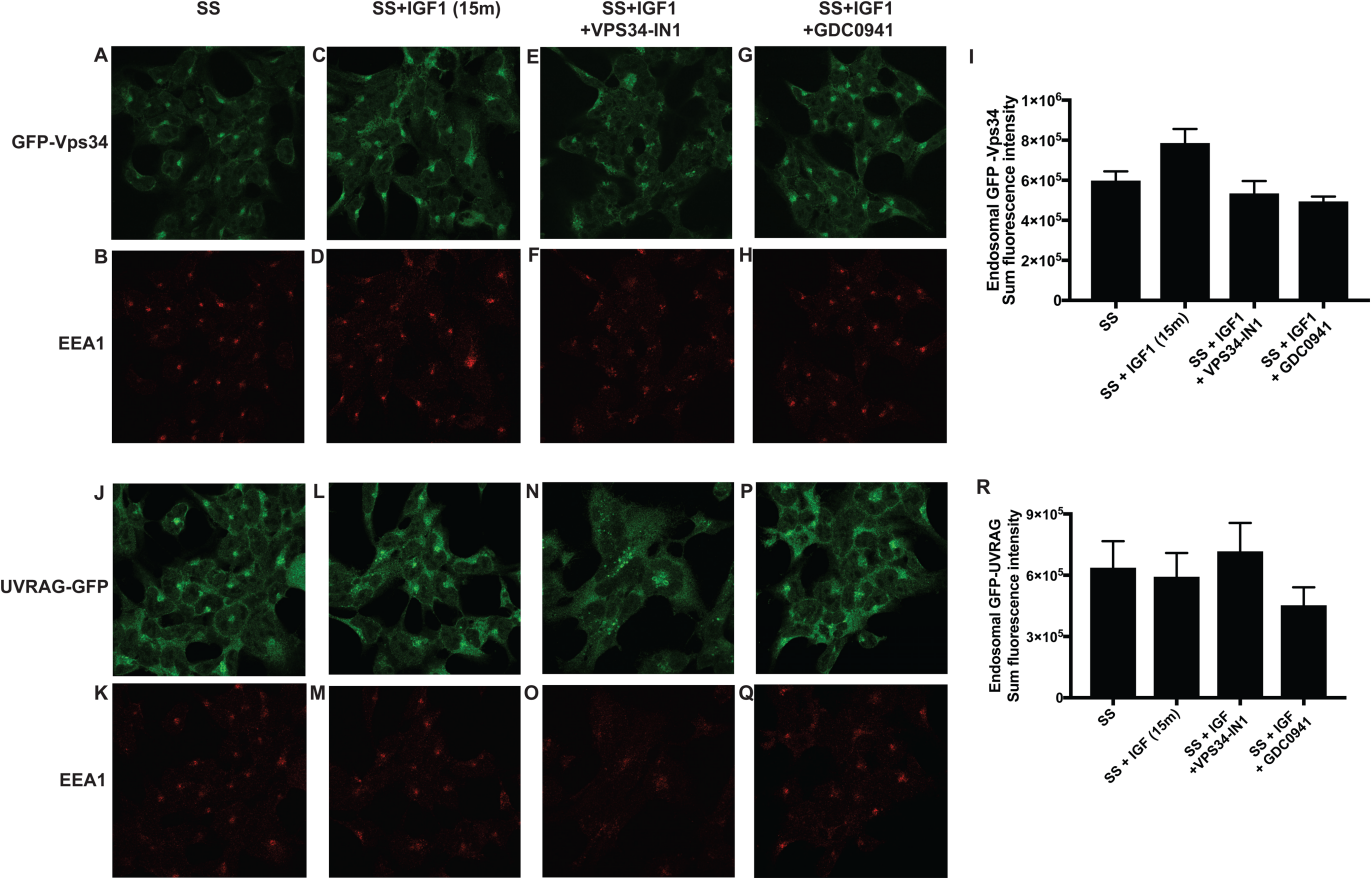
IGF1 does not affect localisation of hVPS34 and UV-RAG. GFP-Vps34 (**A**–**I**) or GFP-UV-RAG (**J**–**R**) knock-in HEK293 cells were serum-starved overnight and treated as indicated with or without VPS34-IN1 (1 µM) and/or GDC-0941 (0.5 µM) for 60 min prior to stimulation with IGF1 for 15 min. Cells were subsequently fixed with 4% (v/v) paraformaldehyde and GFP distribution was visualised using chicken anti-GFP primary and anti-chicken Alexa Fluor 488 to enhance the GFP signal. Co-localisation of hVPS34 or UV-RAG with an early endosomal EEA1 marker was visualised using rabbit anti-EEA1 primary and anti-rabbit Alexa Fluor 594 secondary antibody. The histograms display quantitation of the sum of intensity of fluorescent signal from GFP-Vps34 (**I**) or GFP-UV-RAG (**R**), co-localising with the EEA1 marker ± SEM following the various treatments.

To better study the hVPS34 complex, we employed a CRISPR/CAS9 approach to generate homozygous knock-in mutations in which GFP was fused to the hVPS34 catalytic subunit (N-terminus) as well as the UV-RAG subunit (C-terminus). The fusion protein was expressed at a similar level to that of non-tagged protein in wild-type cells (Figure 5A). Endogenous GFP-VPS34 and GFP-UV-RAG co-localised with EEA1 at the endosome in serum-starved cells (Figure 6). Stimulation with IGF1 for 15 min did not significantly alter endosomal localisation of hVPS34 or UV-RAG. VPS34-IN1 treatment, however, induced a change in morphology of EEA1 and GFP-VPS34 (Figure 6E,F)- and GFP-UV-RAG (Figure 6N,O)-associated endosomal structures. GDC-0941 had no effect on localisation of any hVPS34 component (Figure 6G,H,P,Q).

We also immunoprecipitated endogenous GFP-tagged hVPS34 and UV-RAG employing the high-affinity GFP ‘Trap’ antibodies. Immunoblot analysis confirmed that similar levels of Beclin-1 and UV-RAG were immunoprecipitated with GFP-hVPS34 and GFP-UV-RAG (Figure 5B). We consistently observed that ∼3-fold higher levels of hVPS34 and VPS15 were co-immunoprecipitated with GFP-VPS34 compared with GFP-UV-RAG. The immunoprecipitates were subjected to a PI3-kinase assay and formation of ^32^P-PtdIns(3)P was assessed by thin layer chromatography and autoradiography. As expected, when GFP immunoprecipitations were undertaken from wild-type HEK293 cells not expressing GFP-tagged hVPS34 subunits, no PI3-kinase activity was detected (Figure 5B). Significant PI3K activity was detected in the immunoprecipitates derived from GFP-VPS34 and GFP-UV-RAG that was sensitive to VPS34-IN1 (Figure 5B). Stimulation of cells with IGF1 conditions that activate SGK3 over 10-fold did not significantly affect PI3K activity measured in our assays (Figure 5B).

### Evidence that the UV-RAG complex plays a role in IGF1-induced activation of SGK3

**Figure 7.**
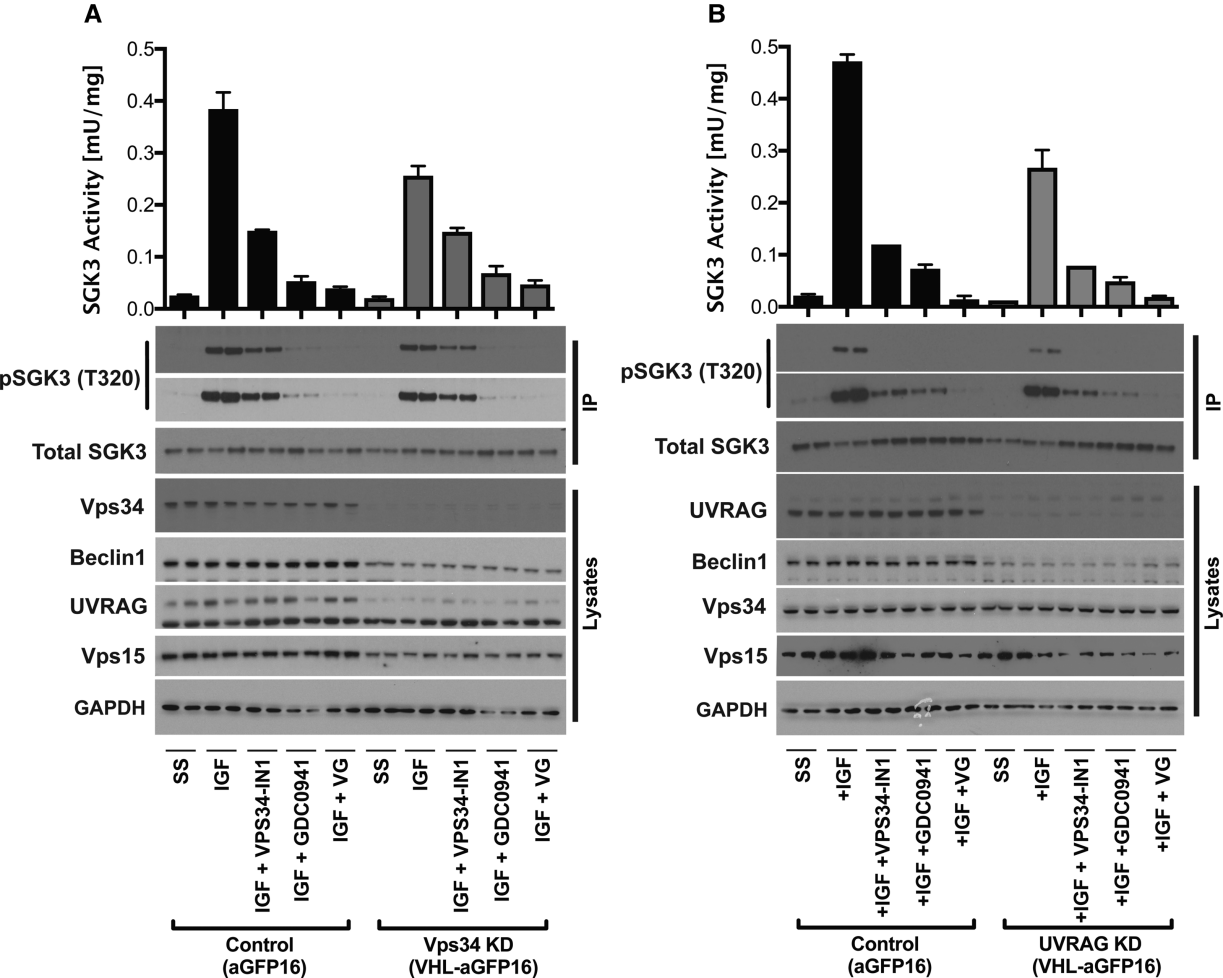
AdPROM-mediated knock-down of hVPS34 and UV-RAG reduces IGF1-induced SGK3 activation. GFP-Vps34 (**A**) or GFP-UV-RAG (**B**) knock-in HEK293 cells that stably express either the anti-GFP nanobody (Control-aGFP16) or the VHL-E3-ligase–anti-GFP nanobody (VHL-aGFP16) were generated as described in the Materials and Methods. Cells were serum-starved overnight and treated as indicated with or without VPS34-IN1 (1 µM) and/or GDC-0941 (0.5 µM) for 60 min prior to stimulation with 50 ng/ml IGF1 for 15 min. Endogenous SGK3 was immunoprecipitated from cell lysates and kinase activity was assessed by measuring phosphorylation of the Crosstide substrate peptide in the presence of 0.1 mM ^32^PγATP in a 30 min reaction. Both immunoprecipitates and lysates were subjected to western blotting with the indicated antibodies. Kinase reactions are presented as means ± SEM for triplicate reactions. Similar results were obtained in at least two independent experiments for all data shown.

To study the impact that knock-down of VPS34 or UV-RAG had on SGK3 activity, we employed a recently described method termed ‘Affinity-Directed Protein Missile System (AdPROM)’ [34,40] to induce ubiquitylation and proteasome-mediated degradation of GFP epitope-tagged proteins. This approach involves use of retroviral overexpression of the VHL1 E3-ligase fused to an anti-GFP nanobody-16 (VHL-aGFP16) that binds and induces degradation of GFP fusion knock-in proteins. Our control cells only expressed the anti-GFP nanobody-16 (aGFP16) (Figure 7). We overexpressed VHL-aGFP16 in the GFP-VPS34 and GFP-UV-RAG knock-in HEK293 cells, which induced ∼90% knock-down of hVPS34 (Figure 7A) or UV-RAG (Figure 7B). Knock-down of hVPS34 also reduced expression Beclin-1, VPS15 and UV-RAG (Figure 7A). Knock-down of UV-RAG had no marked effect on hVPS34 expression, but reduced levels of Beclin-1 and VPS15 (Figure 7B). Our studies reveal that reducing expression of either hVPS34 or UV-RAG by over 90% partially inhibited IGF1-induced activation of SGK3 (Figure 7A,B). A similar reduction in SGK3 was observed in the UV-RAG knock-down compared with VPS34 knock-down (Figure 7A,B). The reduced SGK3 activity in these cells could be further inhibited by VPS34-IN1, indicating that low levels of VPS34 complex still present contribute towards SGK3 activity (Figure 7A,B).

### Role that SHIP2 plays in regulating IGF1-induced activation of SGK3

**Figure 8.**
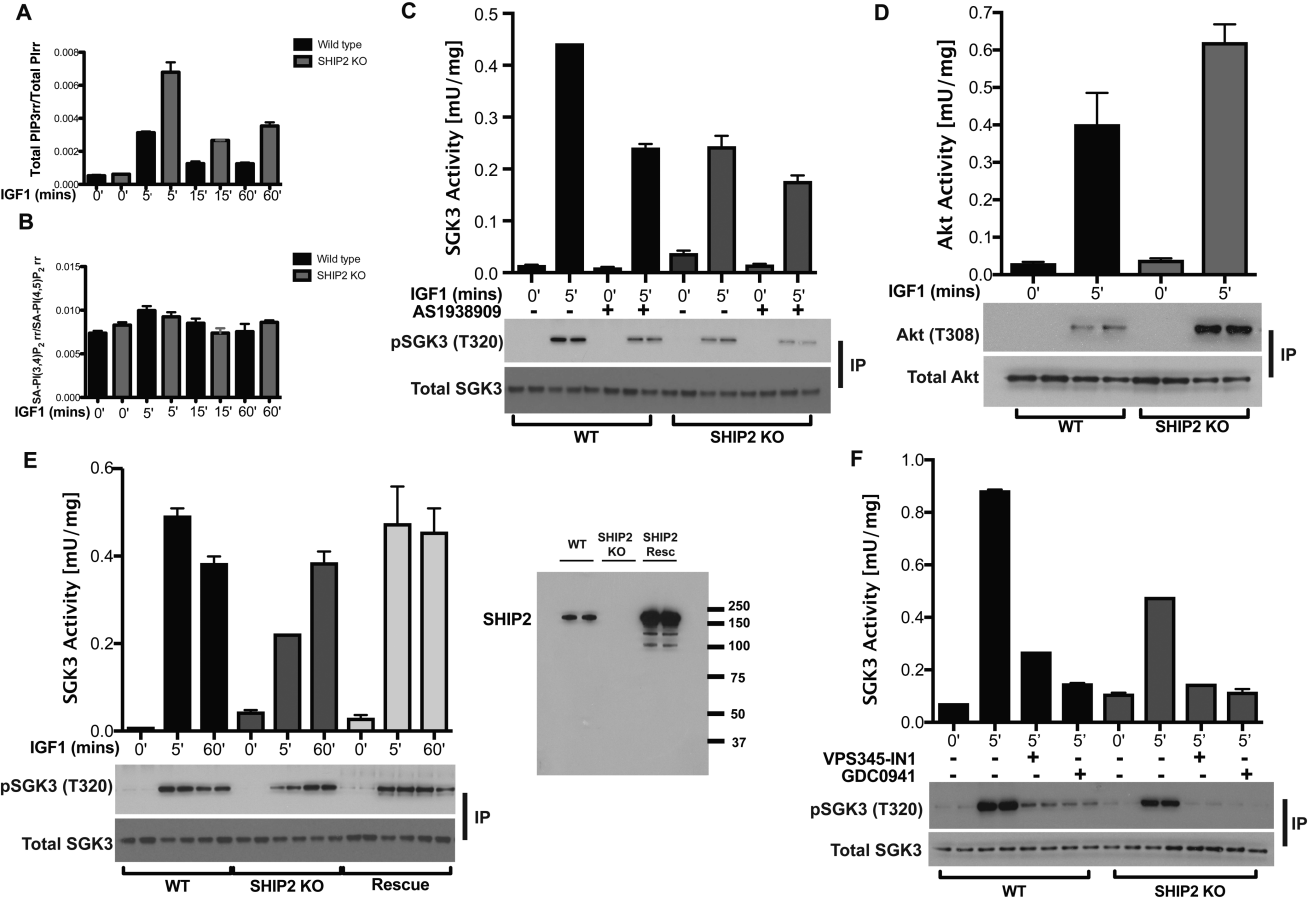
SHIP2 regulates SGK3 activity. (**A** and **B**) Wild-type (WT) or SHIP2 knock-out (KO) cells were serum-starved overnight and stimulated with 50 ng/ml IGF1 stimulation for the indicated time points. Cells were scraped into 0.1 M HCl and snap frozen. PtdIns(3,4,5)P_3_ (**A**) and PtdIns(3,4)P_2_ (**B**) levels were determined. The data are presented for a total of five separate species of PtdIns(3,4,5)P_3_ that were measured. One species of PtdIns(3,4)P_2_ was measured. (**C**) as in (**A**), except that cells were treated in the absence and presence of the SHIP2 inhibitor AS1938909 (10 µM) for 1 h prior to stimulation with IGF1. Endogenous SGK3 was immunoprecipitated from cell lysates with anti-SGK3 antibody and immunoprecipitates subjected to an SGK3 kinase activity assay by measuring phosphorylation of the Crosstide substrate peptide in the presence of 0.1 mM ^32^PγATP in a 30 min reaction. Immunoblot analysis was also carried out on the immunoprecipitates, using the indicated antibodies (**D**). As in (**A**), except that Akt kinase activity was assayed by measuring phosphorylation of the Crosstide substrate peptide in the presence of 0.1 mM ^32^PγATP in a 30 min reaction. Akt immunoprecipitates were subjected to immunoblot analysis with the indicated antibodies. (**E**) As in (**A**), except that HA-SHIP2 was re-expressed in the SHIP2 knock-out (SHIP2 rescue) as described in the Materials and Methods. Wild-type, SHIP2 KO and SHIP2 Rescue cell lysates were immunoblotted with SHIP2 antibody to demonstrate SHIP2 knock-out and rescue in comparison with WT. (**F**) As in (**A**), except that cells were treated with VPS34-IN1 (1 µM) or GDC-0941 (0.5 µM) for 1 h prior to stimulation with 50 ng/ml IGF1 for the indicated times.

As described in the Introduction, PtdIns(3)P can be generated in a hVPS34-independent manner by metabolism of PtdIns(3,4,5)P_3_ to PtdIns(3,4)P_2_ via the SHIP2 phosphatase and then to PtdIns(3)P by INPP4A/B [41]. To explore the contribution that this pathway plays in controlling SGK3 activity, we generated SHIP2 knock-out HEK293 cells by CRISPR/Cas9 methodology and measured PtdIns(3,4,5)P_3_ and PtdIns(3,4)P_2_ levels by a HPLC method. This revealed that, in response to IGF1 stimulation, five different species of PtdIns(3,4,5)P_3_ could be measured that were all elevated 2–3-fold in the SHIP2 knock-out cells at 5, 15 and 60 min following IGF1 stimulation (Figure 8A). The one species of PtdIns(3,4)P_2_ that could be measured was only modestly elevated following 5 min IGF1 stimulation, and did not differ significantly between the wild-type and SHIP2 knock-out cell lines (Figure 8B).

We next measured SGK3 and Akt kinase activity in the wild-type and SHIP2 knock-out cells. After 5 min IGF1 stimulation, despite PtdIns(3,4,5)P_3_ being elevated in SHIP2 knock-out cells, SGK3 activity and T-loop phosphorylation were reduced ∼2-fold compared with those in wild-type cells (Figure 8C). The treatment of wild-type, but not SHIP2 knock-out, cells with the AS1938909 SHIP2 inhibitor [42] also reduced SGK3 activity ∼2-fold (Figure 8C). In contrast, consistent with elevated PtdIns(3,4,5)P_3_, Akt activity and Thr308 phosphorylation were enhanced in the SHIP2 knock-out cells (Figure 8D). Re-introducing SHIP2 into knock-out cells, restored activation of SGK3 and T-loop phosphorylation to a similar extent as that observed in wild-type cells at the 5 min time point of IGF1 stimulation (Figure 8E). At longer time point of 60 min, SHIP2 knock-out did not affect the degree of activation of SGK3 (Figure 8E). We also observed that in SHIP2 knock-out cells, after 5 min IGF1 stimulation when PtdIns(3,4,5)P_3_ levels are elevated (Figure 8A), GDC-0941 still reduces SGK3 activity to near-basal levels (Figure 8F). This observation could be accounted for by a model in which PtdIns(3,4,5)P_3_ promotes SGK3 activity by stimulating mTORC2 and/or by other inositol phosphatases converting PtdIns(3,4,5)P_3_ into PtdIns(3)P.

**Figure 9.**
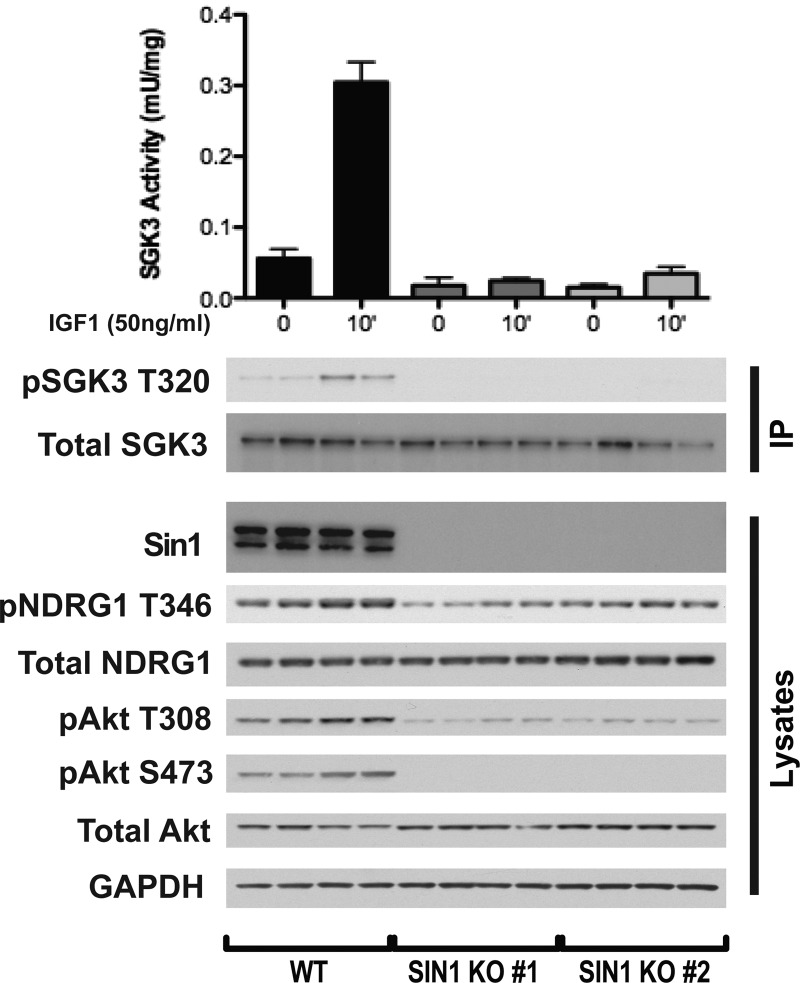
Loss of mTORC2 activity ablates IGF1-induced SGK3 activity. Wild-type and SIN1 knock-out HEK293 cells were serum-starved overnight prior to 50 ng/ml IGF1 stimulation for 15 min. Endogenous SGK3 was immunoprecipitated from the lysates and SGK3 kinase activity was assessed by measuring phosphorylation of the Crosstide substrate peptide in the presence of 0.1 mM ^32^PγATP in a 30 min reaction. Both immunoprecipitates and lysates were subjected to western blotting with the indicated antibodies.

To further explore the possibility that mTORC2 is involved in activating SGK3 by IGF1, we generated a CRISPR/CAS9-mediated knock-out of SIN1 subunit of mTORC2 in HEK293 cells. We observed that loss of mTORC2 activity due to knocking-out of SIN1 also strikingly ablates SGK3 activation by IGF1 (Figure 9). As a control, we also show that deletion of SIN1 also abolishes Akt phosphorylation at Ser473 (Figure 9). These results are consistent with a previous study where it was demonstrated that siRNA knock-down of Rictor subunit of mTORC2 in ZR751 breast cancer cells also inhibited SGK3 activity [17].

**Figure 10.**
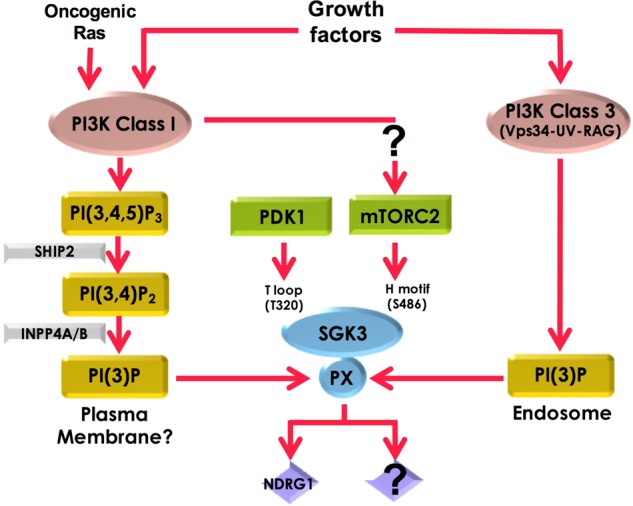
Summary of how SGK3 is activated by growth factors. Our work suggests that three upstream pathways contribute to SGK3 activity. Firstly, IGF1 stimulates production of endosomal PtdIns(3)P probably through the UV-RAG–VPS34 complex, thereby recruiting SGK3 to endosomes where it is activated. Secondly, Class 1 PI3K stimulates SGK3 through enhanced production of PtdIns(3)P resulting from sequential dephosphorylation of PtdIns(3,4,5)P_3_ by SHIP2 and then INPP4A/B inositol phosphatases. It is not clear where PtdIns(3)P generated through this route resides. Thirdly, our data are consistent with a model in which PtdIns(3,4,5)P_3_, produced from activation of Class 1 PI3K, promotes phosphorylation of the hydrophobic motif of SGK3 by mTORC2, thereby stimulating activation of SGK3 by PDK1.

## Discussion

We demonstrate that various growth factors including IGF1 and EGF as well as insulin activate SGK3. Inhibiting either Class 1 or Class 3 PI3K with selective inhibitors partially reduces SGK3 activity. A combination of these inhibitors is needed to ablate SGK3 activity. The inhibitors used GDC-0941 (Class 1 PI3K) [38] and VPS34-IN1 (Class 3 PI3K) [6] are highly selective and do not inhibit Class 2 PI3Ks, indicating that these do not play a major role in controlling growth factor-mediated activation of SGK3. These results establish that both Class 1 and Class 3 PI3K pathways are needed to maximally activate SGK3. However, after inhibiting either Class 1 or Class 3 PI3K, the remaining 20–40% SGK3 activity is still sufficient to phosphorylate NDRG1 to almost the same extent as in the absence of inhibitors (Figure 2). It is possible that there are physiological situations or stresses that lead to the inhibition of Class 1 or Class 3 PI3K pathways, in which SGK3 has evolved to remain active whenever one of these pathways is substantially suppressed.

To investigate how IGF1 controls PtdIns(3)P levels and in turn SGK3, we monitored binding of the HRS2xFYVE PtdIns(3)P binding probe and studied localisation of endogenous knock-in GFP-SGK3 in permeabilised fixed cells (Figure 4). We found that IGF1 induced ∼2-fold increase in endosomal PtdIns(3)P in a manner that was blocked with hVPS34. This is consistent with IGF1 activated SGK3 by enhancing endosomal PtdIns(3)P levels by stimulating the activity of hVPS34. Earlier studies have also reported that insulin moderately enhances PtdIns(3)P levels in adipocytes, myocytes, fibroblasts and hepatocytes [43–46]. We observed that PtdIns(3)P, even after stimulation with IGF1, was mainly localised to endosomal structures and not observed at the plasma membrane, findings consistent with most previous work [6,47–49]. However, treatment with the PI3K Class I inhibitor GDC0941 did induce an ∼2-fold reduction in total PI(3)P levels in cells. It should be noted that one study reported that insulin increased PtdIns(3)P in the plasma membrane in adipocytes [43] and another at lamellipodia [46], based on the use of the same HRS2xFYVE probe utilised in our work. However, we are not able to confirm these results in HEK293 cells analysed under conditions in which SGK3 is activated via the hVPS34 pathway. To more easily study the intracellular localisation and immunoprecipitate endogenous hVPS34 complex, we generated knock-in mutations in which hVPS34 or UV-Rag was endogenously tagged with GFP. Localisation studies confirmed that hVPS34 and its regulatory UV-RAG subunit are localised at the endosomal membranes, consistent with much previous work [50,51]. Following IGF1 stimulation, we did not observe obvious spatial changes in the endosomal localisation of hVPS34 or UV-RAG components. This does not rule out that there is a small, spatially restricted pool of IGF1-regulated PtdIns(3)P and hVPS34–UV-Rag complex that is targeted to discrete mircodomains that cannot be resolved by the analysis that we have undertaken.

Following immunoprecipitation of endogenous GFP-VPS34 or GFP-UV-RAG, we observed PI3K activity that could be inhibited with VPS34-IN1. However, following IGF1 stimulation, we did not observe stimulation of PI3K activity in our immunoprecipitation experiments. It is possible that only a small localised pool of hVPS34 is activated by IGF1 that cannot be measured over the background of total hVPS34 activity that was immunoprecipitated. We also cannot rule out that the IGF1-activated pool of hVPS34 in cells may not be stable to immunoprecipitation or that a factor required for activation is missing in our *in vitro* assays. One previous study reported after immunoprecipitation of the UV-RAG complex of VPS34 from insulin-stimulated primary hepatocytes that a 1.4-fold increase in PI3K activity was observed after 30 min that increased to 2-fold after 60 min insulin stimulation [45]. The reported activation of UV-RAG-immunoprecipitated PI3K activity observed in the present study was much slower than the time course required for IGF1 to enhance SGK3 (maximally 2–5 min). We immunoprecipitated the UV-RAG complex in seven separate experiments and failed to observe a significant stimulation of activity even at the 60 min time point (Figure 5).

As knock-down of the UV-RAG subunit of hVPS34 complex induces the same loss of SGK3 activity than the equivalent knock-down of hVPS34 (Figure 7), we conclude that the UV-RAG complex of hVPS34 plays a role in controlling IGF1 stimulation of SGK3. We observe that ∼90% knock-down of hVPS34 or UV-RAG only partially reduces SGK3 activity (compared with the treatment with hVPS34 inhibitor), emphasising the high sensitivity of the system and that only a small proportion of the total VPS34/UV-RAG complex is required to activate SGK3. This further highlights how SGK3 may have evolved to counteract inhibition of Class 3 PI3K and remains active under conditions where hVPS34 is substantially inhibited. Further work is required to establish how growth factors regulate the activity and/or localisation of the UV-RAG complex of hVPS34 to enhance PtdIns(3)P levels and hence SGK3 activity. In this regard, previous studies have noted that UV-RAG is phosphorylated by mTORC1 [32,52] and possibly Akt [53]. It would be interesting to explore whether phosphorylation of UV-RAG contributed to the mechanism by which growth factors stimulate hVPS34.

Regarding the mechanism by which Class 1 PI3K controls SGK3, our data are in agreement with previous work [6,41], indicating that PtdIns(3,4,5)P_3_ generated through activation of Class 1 PI3K is converted into PtdIns(3)P via the sequential actions of inositol phosphatases SHIP2 [14] and phosphatidylinositol 4-phosphatases (INPP4A/B) [15]. Knock-out or inhibition of SHIP2 would be expected to lower the level of PtdIns(3)P resulting from dephosphorylation of PtdIns(3,4,5)P_3_ and therefore reduce SGK3 activity; this is what we observe at an early 5 min IGF1 treatment time point (Figure 8). It is well established that PtdIns(3,4,5)P_3_ and PtdIns(3,4)P_2_ are mainly located within the plasma membrane where Class 1 PI3K is activated [49,54]. We would therefore expect that the pool of PtdIns(3)P generated through dephosphorylation of PtdIns(3,4,5)P_2_ would also reside on the plasma membrane. However, we were unable to visualise significant PtdIns(3)P or SGK3 at the plasma membrane, even after IGF1 stimulation (Figure 4). The reasons for this are not clear and could arise from plasma membrane concentrations of PtdIns(3)P or GFP-SGK3 being too low or transient to detect using the technology we are deploying. Other groups have also reported that SGK3 resides at the endosome and cannot be visualised at the plasma membrane [41,55]. We cannot rule out that PtdIns(3)P produced by from dephosphorylation of PtdIns(3,4,5)P_3_ is somehow localised elsewhere in cells rather than the plasma membrane resulting from some unknown membrane trafficking pathway. In this regard, we noted that treatment of cells with the Class 1 PI3K inhibitor GDC-0941 moderately reduced the signal from the HRS2xFYVE probe (Figure 4I). Future work is required to address where PtdIns(3)P produced from the dephosphorylation of PtdIns(3,4,5)P_3_ resides and activates SGK3.

Class 1 PI3K is thought to also contribute to the activation of mTORC2 [56], with one study suggesting that this is mediated through PtdIns(3,4,5)P_3_-binding to the PH domain of the SIN1 component of mTORC2 [57]. Stimulation of mTORC2 activity through Class 1 PI3K would also be expected to lead to hydrophobic motif phosphorylation of SGK3 triggering it to be activated by PDK1 [5]. This could represent a third upstream pathway by which SGK3 activity is controlled. Our observations that Class 1 PI3K inhibitors still suppress SGK3 activity in SHIP2 knock-out cells that have elevated PtdIns(3,4,5)P_3_ levels support a model in which Class 1 PI3K is promoting mTORC2 to activate SGK3. However, we cannot rule out that in the SHIP2 knock-out cells, other inositol phosphatases convert PtdIns(3,4,5)P_3_ into PtdIns(3)P, explaining why Class 1 inhibitors still supress SGK3 activity. Oncogenic Ras is well known to activate Class 1 PI3K [58], but there are no reports on whether it is also capable of activating Class 3 PI3K. We find that overexpression of oncogenic Ras leads to activation of SGK3 that, unlike IGF1 stimulation, is entirely inhibited by treatment with Class 1 PI3K inhibitors. This demonstrates that oncogenic Ras in contrast with growth factors is unable to activate Class 3 PI3K and emphasises that activation of SGK3 could contribute to Ras-driven tumour biology.

In summary, we demonstrate that in response to growth factors such as IGF1, three upstream pathways contribute to SGK3 activity (Figure 9). Firstly, IGF1 stimulates production of endosomal PtdIns(3)P probably through the UV-RAG VPS34 complex, thereby recruiting SGK3 to endosomes where it is activated. More work is needed to define the mechanism by which IGF1 controls the UV-RAG complex of hVPS34. Secondly, Class 1 PI3K stimulates SGK3 through enhanced production of PtdIns(3)P resulting from dephosphorylation of PtdIns(3,4,5)P_3_. Additional work is needed to define where the PtdIns(3)P produced through this pathway resides, and why it cannot be visualised on the plasma membrane. Thirdly, our data are consistent with a model in which PtdIns(3,4,5)P_3_, produced from activation of Class 1 PI3K, promotes phosphorylation of the hydrophobic motif of SGK3 by mTORC2, thereby stimulating activation of SGK3 by PDK1. The results presented in the present study emphasise the diverse pathways that exist in cells to activate SGK3 *in vivo*. They also illustrate how SGK3 is well placed to remain substantially active and counteract physiological conditions or stresses that lead to the inhibition of either Class 1 or Class 3 PI3K pathways.

## Abbreviations

AdPROM: Affinity-Directed Protein Missile System
AGC: protein kinase A, protein kinase B, protein kinase G
EEA1: early endosome autoantigen 1
EGF: epidermal growth factor
ERBB: INPP4A/B, inositol polyphosphate-4-phosphatase type A and B
FGF: fibroblast growth factor
GFP: green fluorescent protein
HA: hemagglutinin
hVPS34: human vacuolar protein sorting 34
IGF: insulin-like growth factor
ISD: internal standard
LKA: lipid kinase assay
mTOR: mammalian target of rapamycin
mTORC2: mTOR complex 2
NDRG1: N-myc downstream-regulated gene-1
PDK1: phosphoinositide-dependent kinase-1
PH: pleckstrin homology
PI3K: phosphoinositide 3-kinase
PRAS40: proline-rich AKT substrate 40 kDa
PtdIns: phosphatidylinositol
PX: phox homology
SHIP2: SH2-containing inositol 5′-phosphatase
SGK3: serum and glucocorticoid-regulated kinase-3
