# Supplementary material for: Mechanism of activation of SGK3 by growth factors via the Class 1 and Class 3 PI3Ks
Source: Biochem J. 2018 Jan 2;475(1):117–35. doi: 10.1042/BCJ20170650 (PMC5748840; doi:10.1042/BCJ20170650)
Supplement: Supplementary Figure S1 [file BCJ-475-117-s1.pdf]

Figure S1

A

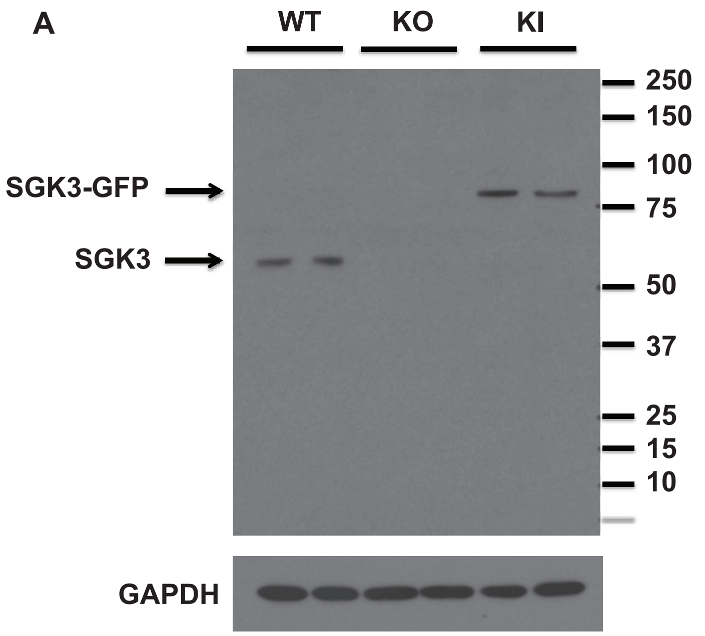

WT
KO
KI
250
150
100
75
50
37
25
15
10
SGK3-GFP
SGK3
GAPDH

B

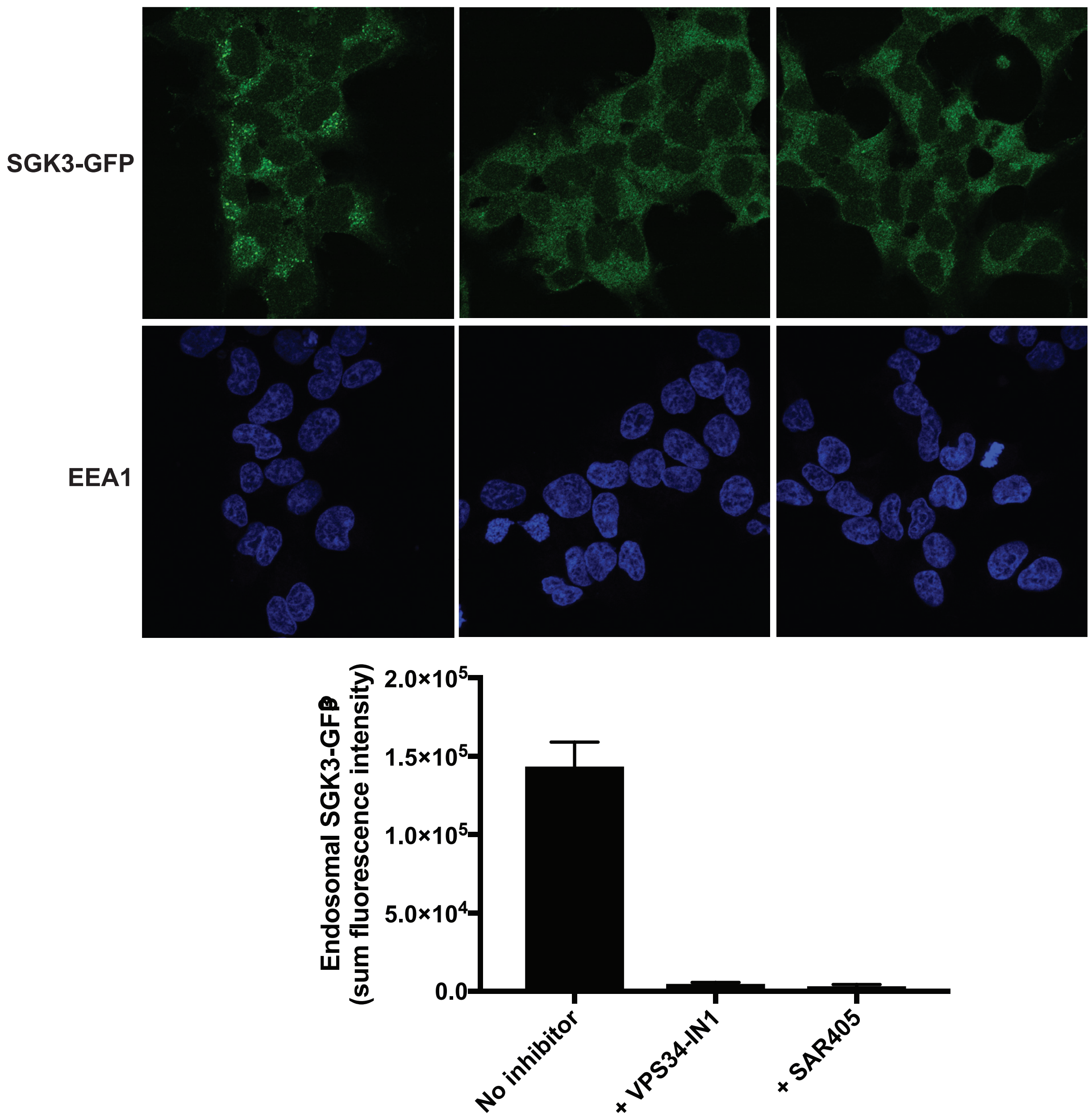

No inhibition
+VPS34-IN1
+SAR405
SGK3-GFP
EEA1
Endosomal SGK3-GFP
(sum fluorescence intensity)
2.0×10^5
1.5×10^5
1.0×10^5
5.0×10^4
0.0
No inhibitor
+ VPS34-IN1
+ SAR405

Figure S1 – Generation of SGK3 knock out and GFP-SGK3 knock in cell lines and the impact of hVPS34 inhibition on GFP-SGK3 localisation. (A) Wild type (WT), SGK3 knock-out (KO) and GFP-GFP-SGK3 knock-in (KI) HEK293 cells were lysed and cell lysates subjected to immunoblot analysis with the indicated antibodies. **(B)** GFP-SGK3 knock-in HEK293 cells cultured in 10% (v/v) serum were treated with or without 1µM VPS34-IN1 or 1µM SAR405 for 1 hour prior to fixation with 4% (v/v) paraformaldehyde. GFP distribution in cells was visualized using chicken anti-GFP primary and anti-chicken Alexa Fluor-488 to enhance the GFP signal (upper panel). DAPI was used to stain nuclei (lower panel). Histogram represents quantitation of GFP-SGK3 on endosomal structures using sum of intensity of fluorescent signal from staining of GFP ±S.E.M. following the various treatments.
